# Head CT for Minor Head Injury Presenting to the Emergency Department in the Era of Choosing Wisely

**DOI:** 10.5811/westjem.2017.6.33685

**Published:** 2017-07-12

**Authors:** John DeAngelis, Valerie Lou, Timmy Li, Henry Tran, Praneeta Bremjit, Molly McCann, Peter Crane, Courtney M.C. Jones

**Affiliations:** * University of Rochester Medical Center, Department of Emergency Medicine, Rochester, New York; † Cambridge Health Alliance, Department of Emergency Medicine, Cambridge, Massachusetts; ‡ Jefferson Hospital, Department of Emergency Medicine, Philadelphia, Pennsylvania

## Abstract

**Introduction:**

The Choosing Wisely campaign currently recommends avoiding computed tomography (CT) of the head in low-risk emergency department (ED) patients with minor head injury, based on validated decision rules. However, the degree of adherence to this guideline in clinical practice is unknown. The objective of this study was to evaluate adherence to the Choosing Wisely campaign’s recommendations regarding head CT imaging of patients with minor head injury in the ED.

**Methods:**

We conducted a retrospective cohort study of adult ED patients at a Level I trauma center. Patients aged ≥ 18 years who presented to the ED with minor head injury were identified via International Classification of Diseases, 9th Revision, Clinical Modification codes. Medical record abstraction was conducted to determine the presence of clinical symptoms of the NEXUS II criteria, medical resource use, and head CT findings. We used descriptive statistics to characterize the study sample, and proportions were used to quantify guidelines adherence.

**Results:**

A total of 489 subjects met inclusion criteria. ED providers appropriately applied the Choosing Wisely criteria for 75.5% of patients, obtaining head CTs when indicated by the NEXUS II rule (41.5%), and not obtaining head CTs when the NEXUS II criteria were not met (34.0%). However, ED providers obtained non-indicated CTs in 23.1% of patients. Less than 2% of the sample did not receive a head CT when imaging was indicated by NEXUS II.

**Conclusion:**

ED providers in our sample had variable adherence to the Choosing Wisely head-CT recommendation, especially for patients who did not meet the NEXUS II criteria.

## INTRODUCTION

According to a report by the Institute of Medicine, approximately $750 billion of healthcare spending annually results in no benefit to patients in the United States (U.S.). 1 Minor head injury is a common concern prompting emergency department (ED) visits. In 2010 the number of ED visits in the U.S. for traumatic brain injury (TBI) exceeded 2.5 million. 2 It has been estimated that approximately 75% of TBIs are considered mild. 3 Streamlined assessment of patients presenting with minor head injury to identify those who require imaging, in order to further risk stratify the need for neurosurgical management could result in a significant reduction in healthcare spending.

Computed tomography (CT) of the head is commonly used to assess patients presenting to the ED with head injury. Approximately 80 million CTs are performed in the U.S. each year, with approximately one third of these performed in emergency settings. 4 For patients with obvious signs of TBI, such as evidence of skull fracture on physical exam, or neurologic changes, obtaining head CTs has clear benefit, as advanced imaging may be necessary to guide medical and neurosurgical interventions. 5 However, for patients without obvious signs of TBI, the decision to perform a head CT requires more deliberation. Many non-clinical factors influence a provider’s decision to obtain a CT in patients with minor head injury. These include patient expectations, patient and provider anxiety, fear of litigation, fear of missed diagnoses, and desire to expedite diagnoses. 6–8 Conversely, providers may hesitate to order CTs due to concerns such as increased door-to-discharge times, increased length of hospital stay, harm and cost from incidental findings on imaging, and risk of cancer due to exposure to ionizing radiation.^9^,^10^ A balanced approach is required to ensure ordering of head CTs when necessary, while mitigating the potential downsides of over-imaging.

The American Board of Internal Medicine Foundation launched the Choosing Wisely initiative in 2012 with the goal of advancing dialogue about avoiding wasteful or unnecessary medical tests and procedures. 11 The American College of Emergency Physicians (ACEP) joined this group with five recommendations, one of which is to “[a]void computed tomography (CT) scans of the head in emergency department patients with minor head injury who are at low risk based on validated decision rules.” 12 Many patients with minor head injuries receive unnecessary CTs in the ED that provide no clinical benefit. In an era of increasing medical expenditures, growing ED wait times, and concern for cancers caused by excessive diagnostic radiation exposure, the Choosing Wisely campaign attempts to improve care and decrease costs by avoiding unnecessary testing. However, there is sparse evidence regarding actual rates of adherence to the Choosing Wisely campaign recommendations on avoiding head CTs in low-risk patients.

Population Health Research Capsule

What do we already know about this issue?

Several validated decision rules are available to determine the need for head CTs in minor trauma, and we know that application of these rules can reduce unnecessary CT use.

What was the research question?

To what extent are providers using decision rules for CT use in minor head trauma in light of the Choosing Wisely ACEP guidelines?

What was the major finding of the study?

While application to a decision rule was quite good, there was a portion of head CTs that could have been avoided through application of a CT decision rule.

How does this improve population health?

Increasing awareness about Choosing Wisely and demonstrating clear benefits to the broad application of a CT decision rule in minor head trauma could continue to reduce CT use.

Choosing Wisely cites the Canadian Computed Tomography Head Rule (CCHR) and the New Orleans Criteria as validated decision-making tools used to identify low-risk patients for whom CT head imaging may be safely avoided. Another widely used validated decision–making tool is the NEXUS (National Emergency X-Ray Utilization Study) II rule. 13–17 The NEXUS II rule has been shown to have the highest reduction rate for CTs, with comparable sensitivities and specificities in identifying clinically important brain injury. The NEXUS II criteria also largely match those of the 2008 ACEP clinical policy regarding use of CTs in head trauma patients with no loss of consciousness or post-traumatic amnesia. 18 In addition, the NEXUS II rule consists of binary criteria, an added convenience and advantage in our study design using standardized medical record review and data abstraction. In summary, we chose to use the NEXUS II rule due to its general consistency with other validated decision rules and ACEP clinical policy, acceptable sensitivity and specificity in identifying clinically significant head trauma, convenience of binary criteria in chart review, as well as its ease of application in the ED setting. To evaluate whether common ED practice aligns with Choosing Wisely recommendations, we performed chart reviews on a sample of ED patients with minor head injury to determine if they met NEXUS II criteria, and if they received head CTs. Our first aim was to describe adherence to the NEXUS II rule by determining the proportion and level of agreement between patients who received a CT of the head and whether or not the CT was indicated by the NEXUS II guidelines. Secondly, we aimed to describe physician non-adherence to the NEXUS II guidelines by determining the proportion of patients for whom a CT was not indicated and not obtained compared to patients for whom a head CT was not indicated but obtained. Lastly, we evaluated on a case-by-case basis characteristics of patients for whom a head CT was indicated by the NEXUS II guidelines but not obtained.

## METHODS

### Study Design

This was a retrospective medical record review study of patients presenting to the University of Rochester Medical Center’s Strong Memorial Hospital’s ED between January 1, 2013 and December 31, 2013.

### Study Setting and Population

The Strong Memorial Hospital ED treats over 100,000 patients annually, is the region’s tertiary academic medical center, and is an American College of Surgeons-verified Level I trauma center. The institution’s Research Subjects Review Board approved the conduct of this study with a waiver of informed consent.

### Study Protocol

We queried the ED electronic medical record (EMR) system (for patients (age ≥18 years) with minor head injury using *International Classification of Diseases, 9th Rev., Clinical Modification* (ICD-9-CM) external cause of injury codes. Specific codes used in participant selection were the following: *959.01* (Head injury, not otherwise specified); *850.0–850.9* (Head injury, with and without loss of consciousness); *920.0* (Head contusion); and *873.0–873.9* (Scalp laceration). We selected a random sample of subjects from the initial query. (See sample size calculation under Statistical Analysis.)

We excluded patients if there was an inappropriate application of an ICD-9-CM code or if there was no documentation of head injury to correspond with the ICD-9-CM code (e.g., chief complaint of dental pain). Subjects were also excluded if application of the NEXUS II rule was inappropriate, defined as patients who were at high risk of severe head injury and CT was warranted based on initial ED presentation, or the presence of any of the following: 1) alcohol intoxication; 2) moderate or severe head injury (GCS <14); 3) trauma team activation; or 4) physician ordering a “Multi CT scan”

We conducted a standardized medical record review on all subjects. A data collection form was created with a corresponding data abstraction guide. The data abstraction guide defined each of the variables to be abstracted, including specific details for how to abstract the variable and where in the EMR each variable should be located. The data collection form and abstraction guide were developed through an iterative process with the physician-abstractors (JD, VL, HT, PB). All abstractors collected data concurrently and met regularly to discuss questions, and discrepancies were resolved via consensus review with the investigative team.

### Measurements

**Figure 1:**
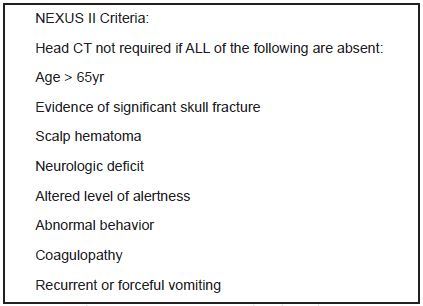
National Emergency X-Ray Utilization Study (NEXUS II) is a validated decision-making tool to aid in determining if computed tomography is necessary in cases of head trauma.

Variables abstracted included patient demographics, presenting chief complaint, symptoms including those outlined by the NEXUS II guidelines (Figure 1), whether or not a head CT was obtained and the corresponding results of the scan, neurosurgical interventions, and ICD-9-CM codes. We performed a review of nursing, resident, advanced practice provider, and attending notes, updated medication lists, medical history, and laboratory results linked to the relevant patient encounter to determine whether components of the decision rule were present for each study subject.

### Data Analysis

We used descriptive statistics to describe the study sample, including patient demographics, presenting neurological symptoms, and CT use. Our primary objectives were to describe adherence to ACEP Choosing Wisely imaging recommendations using NEXUS II as our validated decision rule and determine the extent to which ED providers deviated from this rule. We classified subjects into one of two groups according to the NEXUS II criteria: 1) head CT indicated; and 2) head CT not indicated. These two groups were further stratified based on whether the ED provider actually ordered and obtained a head CT: 1) head CT obtained; and 2) head CT not obtained. Due to the paired nature of the data, a McNemar’s test and Cohen’s kappa coefficient were calculated to determine the extent of agreement between the NEXUS II indications for head CT vs. physician order for head CT.

Our secondary objective was to describe provider non-adherence to the NEXUS II guidelines. We compared demographic and clinical characteristics in subjects for whom a head CT was not indicated and not obtained with subjects for whom a head CT was not indicated but obtained. We used chi-square tests and Fisher’s exact test where appropriate. This comparison allowed us to evaluate whether certain subgroups were subject to higher risk of provider non-adherence to the NEXUS II rule. Among patients for whom head CTs were not indicated but were obtained, we determined the proportion of those with significant findings on head imaging and described the nature of these findings (e.g., depressed skull fracture, intracranial hemorrhage). Additionally, we determined whether these injuries resulted in any neurosurgical intervention (e.g., intracranial pressure monitoring). Thirdly, we categorized the characteristics of those subjects for whom a head CT was indicated by the NEXUS II rule, but was not obtained.

The sample size for the current study was based on a McNemar’s test. We needed 783 subjects to estimate the proportion of subjects for whom the provider adhered to the NEXUS II guidelines with 80% power and type I error of 5%. We conservatively estimated the discordance between NEXUS II-indicated head CT vs. actual provider order for head CT as 10% in the CT indicated but not obtained group, and 15% in the CT not indicated but obtained group. Based on previous experience, we anticipated that a considerable number of subjects would present with alcohol intoxication and subsequently be excluded after the EMR review was initiated. To account for this, as well as other potential exclusions, missing data and incomplete records, we oversampled by a factor of 25%. As such, we began our standardized medical record review with 1,000 randomly selected subjects from the initial pool of patient encounters meeting inclusion criteria.

## RESULTS

**Figure 2:**
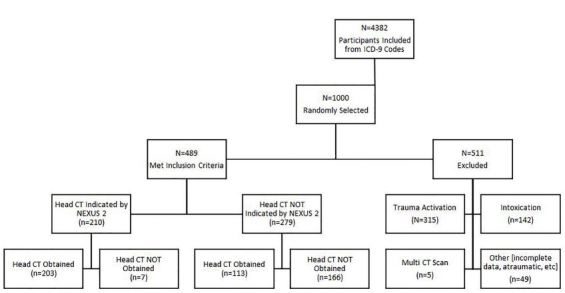
Eligibility criteria for inclusion in study examining providers’ adherence to (computed tomography ) CT decision rules in minor head injury.

The initial medical record query resulted in 4,382 cases of minor head injury that met our ICD-9-CM criteria for inclusion in the study. Of the 1,000 randomly selected participants, 489 met eligibility criteria (Figure 2). The majority of the sample was less than 65 years of age (78.1%), male (54.6%), self-identified as White (76.9%), and of non-Hispanic origin (94.3%) (Table 1). Four patients showed evidence of a skull fracture on physical exam (0.8%), and 104 patients presented with a scalp hematoma (21.3%). Fifteen patients had a neurological deficit (3.1%), 35 exhibited abnormal behavior (7.2%), and 14 experienced excessive or recurrent vomiting (2.9%).

Emergency physicians appropriately applied NEXUS II criteria in 75.5% of subjects (Table 2). Head CTs were obtained when indicated for 203 patients (41.5%). Conversely, head CTs were not obtained when the criteria were not met for 166 patients (33.9%). However, ED providers obtained non-indicated CTs in 23.1% of patients who did not meet the NEXUS II criteria (113 patients). Cases where CTs were indicated by NEXUS II but were not obtained occurred in seven patients (1.4%). Overall, there was a statistically significant difference in the pattern of indicated head CTs vs. obtained head CTs with a kappa coefficient of 0.51 (95% confidence interval [CI] [0.46–0.60]). This is indicative of fair adherence to the NEXUS II criteria. Of those for whom CTs were obtained in non-indicated situations (113 patients), only two revealed significant head injury, and none required neurosurgical intervention.

Table 3 shows the characteristics of patients who did and did not receive a head CT among those for whom a head CT was not indicated. There were no statistically significant demographic or clinical differences, with the exception of patient sex: 55.8% were female and 44.3% were male (p=0.0002). Of the seven encounters where CTs were indicated but not obtained, four patients had documented hematomas, one was on an anti-platelet agent, and three were over the age of 65. These patients should have had CT head imaging in accordance with NEXUS II criteria (Table 4). All seven subjects had low-energy traumatic mechanisms, and none returned to the hospital for the same injury.

## DISCUSSION

Despite evidence suggesting that the use of validated clinical decision rules can be used to identify patients with minor head injuries in whom it is safe to forgo a CT of the head, the use of CT is still widespread among this low-risk patient population. Adherence to the 2012 Choosing Wisely recommendation to avoid head CT in ED patients with minor head injury who are at low risk for TBI based on validated decision rules was unknown. By retrospectively applying NEXUS II, a validated decision rule, to a sample of patients with minor head injury, we aimed to assess adherence to the Choosing Wisely campaign’s recommendation regarding head CT.

The Choosing Wisely campaign does not specify that any particular decision rule be used in the evaluation of ED patients with minor head injuries. Although the CCHR is the most extensively tested decision rule, with a somewhat higher sensitivity than NEXUS II in identifying injuries that require neurosurgical intervention, the CCHR’s exclusion criteria make it difficult to apply universally. 19 For this, and the aforementioned reasons in the background section, we chose to use the NEXUS II rule instead. As previously stated, the CCHR would have been especially difficult to apply retrospectively in our study sample. For example, the CCHR criteria regarding duration of retrograde amnesia and fall height may not always be documented in the medical record. Furthermore, when applied, the NEXUS II rule has been shown to result in the highest reduction in CTs performed compared to other decision rules. 13

In our study, a considerable number of head CTs were obtained without meeting formal NEXUS II criteria (23.1%). ED providers had variable adherence to the NEXUS II head CT recommendation (kappa coefficient of 0.52). Of the 279 patients for whom head CT was not indicated, CTs were obtained in 113 patients (40.5%), with no discernable change in course of care. This indicates that there is room for improvement in the clinical application of the NEXUS II guidelines. However, as previously noted, the decision to obtain a CT of the head may be influenced by numerous clinical and non-clinical factors.^9^,^10^ Because this was a retrospective study relying on EMR review, the exact reasons for obtaining a head CT are unknown. ED providers may not have adequately documented their thought process, or the factors contributing to their ultimate decision to obtain a CT in the medical record. Therefore, providers in the study at the time of care were free to use any decision rule that they felt appropriate, or a gestalt. There is not currently a policy at our center that emphasizes use of one rule.

An unexpected and concerning finding of our study is that seven patients for whom a head CT was indicated by the NEXUS II rule did not receive one. However, none of these patients appeared to have significant injuries based upon individual chart review. Again, the exact reasons to forgo CT in these patients may be difficult to determine from a chart review. Further, due to small sample size and inability to follow up with some of these patients, their long-term outcomes are unknown. We also recognize that while Choosing Wisely recommends that a decision rule be used, it does not specify which one. Clinicians could have used rules other than NEXUS II and still have complied with the recommendation. We were unable to account for all decision rules and may have missed instances where other rules were applied. Instead we used one that is both commonly applied and conducive to our method of retrospective chart review.

In summary, we found that application of the NEXUS II decision rule in an urban Level I trauma center in accordance with Choosing Wisely recommendations for avoiding imaging in minor head injury remains variable. While it appears that practitioners are using NEXUS II criteria appropriately to indicate the necessity of CT imaging, there is room for improvement in use for avoiding CT imaging. This would support the Choosing Wisely campaign’s stance that physicians can continue to make better clinical decisions that are likely to improve care, perhaps by reducing possibly harmful ionizing radiation, resource utilization, and costs associated with unnecessary imaging tests. While it is true that rules such as NEXUS II, the CCHR, and the New Orleans Criteria have been discussed extensively for the past 10 years, the advent of Choosing Wisely and ACEP’s contribution to its recommendations put these rules into a different context. There is now more incentive to use these rules to protect patients and conserve resources. Therefore, it is important to quantify how the rules were applied both before and after Choosing Wisely was published. Future studies may potentially examine head-injured patients who are under the influence of alcohol, since almost 50% of our initial sample was excluded due to its presence.

## LIMITATIONS

Although we started with 1,000 patients, more than 50% were excluded. The reasons for their exclusion are outlined in Figure 2. We believe that these exclusions were appropriate and necessary to address our research question in the most rigorous way possible.

Less than 2% of our sample did not receive a head CT when one was indicated by the decision rule, limiting our ability to accurately describe this population. A larger sample size may be able to better characterize these subjects. The frequency of indicated but non-obtained head CTs is likely low in actuality, but does warrant future evaluation.

We also recognize that by identifying patients through the use of ICD-9-CM codes we may have missed patients with minor head injuries who may have otherwise been qualified for inclusion into our study. It is unclear how or if these patients would differ with respect to meeting the clinical decision rules and obtaining head CTs. In addition, the total number of patients with minor head trauma may be an underestimate. A different ICD-9-CM code may have been assigned after NEXUS II criteria resulted in a CT and intracranial hemorrhage was identified.

The proportion of subjects who met the decision rule is dependent on the accuracy of medical record documentation as well as data abstraction. We attempted to mitigate potential inaccuracies through our choice of NEXUS II for the decision rule, as the individual criteria outlined in this rule are frequently and consistently documented in our EMR. We also developed a detailed data abstraction guide and performed consensus review on any questionable data fields for specific cases. However, as previously addressed in our discussion, we could not control for the use of this rule alone. Clinicians may have used any decision rule or gestalt at time of care, which introduces unknown bias into the results.

The retrospective nature of our study was not ideal for determining adherence to a specific decision rule. A blinded prospective study in which all providers were instructed to use only NEXUS II in determining whether to perform head CTs for minor head trauma would have been ideal. However, the importance of quantifying adherence to these decision-making rules only became apparent after Choosing Wisely was published.

Lastly, we acknowledge that practice patterns differ significantly across regions. As seen in other reviews, such as the Dartmouth Atlas, 20 our experience in a single trauma center may not be representative of practice patterns at other institutions. As such, the external validity of our findings should be confirmed in future research and independent samples.

## CONCLUSION

In our sample of patients with minor head injury, ED utilization of head CT aligns with clinical guidelines for the majority of patients. However, a significant proportion of subjects received head CTs when not indicated by NEXUS II criteria. Further investigation of factors that influence physician decision-making surrounding the use of head CTs for patients with minor head injury is warranted.

## Figures and Tables

**Table 1:** Subject characteristics (N=489).

Subject characteristic	N (%)
Age	
≥65	107 (21.9)
<65	382 (78.1)
Sex	
Female	222 (45.4)
Male	267 (54.6)
Race	
American Indian	1 (0.2)
Asian	7 (1.4)
Black	95 (19.4)
Native Hawaiian	1 (0.2)
Other	9 (1.8)
White	376 (76.9)
Ethnicity	
Hispanic or Latino	28 (5.7)
Not Hispanic or Latino	461 (94.3)
Evidence of skull fracture on physical exam	
Yes	4 (0.8)
No	485 (99.2)
Scalp hematoma	
Yes	104 (21.3)
No	385 (78.7)
Neurologic deficit	
Yes	15 (3.1)
No	474 (96.9)
GCS <15	
Yes	19 (3.9)
No	470 (96.1)
Abnormal behavior	
Yes	35 (7.2)
No	454 (92.8)
Platelets <50 10^3^/uL	
Yes	1 (0.2)
No	488 (99.8)
INR >1.5	
Yes	8 (1.6)
No	481 (98.4)
Coagulopathy	
Yes	20 (4.1)
No	469 (95.9)
Recurrent vomiting	
Yes	14 (2.9)
No	475 (97.1)
Anticoagulant medication use	
Yes	11 (2.3)
No	478 (97.7)
Platelet inhibitor use	
Yes	58 (11.9)
No	431 (88.1)

GCS, Glasgow Coma Scale; INR, international normalized ratio.

**Table 2:** Concordance of CT indicated and CT obtained.

	CT obtained		
		
CT indicated	Yes	No	Total
Yes	203	7	210
No	113	166	279
Total	313	176	489
		n	%
			
CT indicated and obtained		203	41.5
CT not indicated and not obtained		166	40.0
Overall concordance		369	75.5
CT indicated and not obtained		7	1.4
CT not indicated and not obtained		113	23.1
Overall discordance		120	24.5

Kappa= 0.5161, 95% CI [0.4619–0.5954].

CT, computed tomography.

**Table 3:** Differences in characteristics of patients not meeting criteria for a head CT, who did and did not receive a CT (n = 279).

	CT not indicated and obtained (n = 113)	CT not indicated and not obtained (n = 166)	
			
	N (%)	N (%)	p value
Age			NS
<65	0 (0.00)	1 (0.60)	
≥65	113 (100.0)	165 (99.4)	
Sex			0.0002
Female	63 (55.8)	55 (33.1)	
Male	50 (44.3)	111 (66.9)	
Race			0.0909
Asian	5 (4.4)	1 (0.6)	
Black	26 (23.0)	41 (24.7)	
Other	5 (4.4)	3 (1.8)	
White	77 (68.1)	121 (72.9)	
Ethnicity			NS
Hispanic or Latino	10 (8.9)	15 (9.0)	
Not Hispanic or Latino	103 (91.2)	151 (91.0)	
Evidence of skull fracture			
No	113 (100.0)	166 (100.0)	
Scalp hematoma			NS
Yes	1 (0.9)	0 (0.0)	
No	112 (99.1)	166 (100.0)	
Neurological deficit			
No	113 (100.0)	166 (100.0)	
GCS <15			
No	113 (100.0)	166 (100.0)	
Abnormal behavior			
No	113 (100.0)	166 (100.0)	
Platelets <50 10^3^/uL			
No	113 (100.0)	166 (100.0)	
INR >1.5			
No	113 (100.0)	166 (100.0)	
Coagulopathy			
No	113 (100.0)	166 (100.0)	
Recurrent vomiting			
No	113 (100.0)	166 (100.0)	
Anticoagulant medication			
No	113 (100.0)	166 (100.0)	
Platelet inhibitor			NS
Yes	3 (2.7)	2 (1.2)	
No	110 (97.3)	164 (98.8)	

GCS, Glasgow Coma Scale; INR, international normalized ratio; CT, computed tomography.

**Table 4:** Characteristics of subjects for whom a head CT was indicated but not obtained (N=7).

Subject characteristic	N (%)
Age	
≥65	3 (42.9)
<65	4 (57.1)
Gender	
Female	5 (71.4)
Male	2 (28.6)
Race	
White	7 (100.0)
Ethnicity	
Not Hispanic or Latino	7 (100.0)
Treating provider level of training	
Resident	1 (14.3)
Other/unknown	6 (85.7)
Evidence of skull fracture	
No	7 (100.0)
Scalp hematoma	
Yes	4 (57.1)
No	3 (42.9)
Neurological deficit	
No	7 (100.0)
GCS<15	
No	7 (100.0)
Abnormal behavior	
No	7 (100.0)
Platelets <50 10^3^/uL	
No	7 (100.0)
INR >1.5	
No	7 (100.0)
Coagulopathy	
No	7 (100.0)
Recurrent vomiting	
No	7 (100.0)
Anticoagulant medication use	
No	7 (100.0)
Platelet inhibitor use	
Yes	1 (14.3)
No	6 (85.7)

GCS, Glasgow Coma Scale; INR, international normalized ratio.

## References

[1] Committee on the Learning Health Care System in America; Institute of Medicine (2013). Best Care at Lower Cost: The Path to Continuously Learning Health Care in America.

[2] Marin JR, Weaver MD, Yealy DM (2014). Trends in visits for traumatic brain injury to emergency departments in the united states. JAMA.

[3] Uhl RL, Rosenbaum AJ, Czajka C (2013). Minor traumatic brain injury: a primer for the orthopaedic surgeon. J Am Acad Orthop Surg.

[4] Sierzenski PR, Linton OW, Amis ES (2014). Applications of justification and optimization in medical imaging: examples of clinical guidance for computed tomography use in emergency medicine. Ann Emerg Med.

[5] Rivara FP, Kuppermann N, Ellenbogen RG (2015). Use of Clinical Prediction Rules for Guiding Use of Computed Tomography in Adults With Head Trauma. JAMA.

[6] Melnick ER, Shafer K, Rodulfo N (2015). Understanding Overuse of Computed Tomography for Minor Head Injury in the Emergency Department: A Triangulated Qualitative Study. Acad Emerg Med.

[7] Probst MA, Kanzaria HK, Schriger DL (2014). A conceptual model of emergency physician decision making for head computed tomography in mild head injury. Am J Emerg Med.

[8] Rohacek M, Albrecht M, Kleim B (2012). Reasons for ordering computed tomography scans of the head in patients with minor brain injury. Injury.

[9] Miglioretti DL, Johnson E, Williams A (2013). The use of computed tomography in pediatrics and the associated radiation exposure and estimated cancer risk. JAMA Pediatr.

[10] Smith-Bindman R, Lipson J, Marcus R (2009). Radiation dose associated with common computed tomography examinations and the associated lifetime attributable risk of cancer. Arch Intern Med.

[11] American Board of Internal Medicine Foundation (2016). Choosing Wisely.

[12] American College of Emergency Physicians (2014). Five Things Physicians and Patients Should Question. Choosing Wisely.

[13] Ro YS, Shin SD, Holmes JF (2011). Comparison of clinical performance of cranial computed tomography rules in patients with minor head injury: a multicenter prospective study. Acad Emerg Med.

[14] Smits M, Dippel DW, de Haan GG (2005). External validation of the Canadian CT Head Rule and the New Orleans Criteria for CT scanning in patients with minor head injury. JAMA.

[15] Mower WR, Hoffman JR, Herbert M (2005). Developing a decision instrument to guide computed tomographic imaging of blunt head injury patients. J Trauma.

[16] Papa L, Stiell IG, Clement CM (2012). Performance of the Canadian CT Head Rule and the New Orleans Criteria for predicting any traumatic intracranial injury on computed tomography in a United States Level I trauma center. Acad Emerg Med.

[17] Stiell IG, Clement CM, Rowe BH (2005). Comparison of the Canadian CT Head Rule and the New Orleans Criteria in patients with minor head injury. JAMA.

[18] Jagoda AS, Bazarian JJ, Bruns JJ (2008). Clinical policy: neuroimaging and decisionmaking in adult mild traumatic brain injury in the acute setting. Ann of Emerg Med.

[19] Harnan SE, Pickering A, Pandor A (2011). Clinical decision rules for adults with minor head injury: a systematic review. J Trauma.

[20] Wennberg JE (2002). Unwarranted variations in healthcare delivery: implications for academic medical centres. BMJ.

